# Heidelberg ETV score to assess success of ETV in patients with occlusive hydrocephalus: a retrospective single-center study

**DOI:** 10.1007/s10143-023-02122-0

**Published:** 2023-08-29

**Authors:** Mohammed Issa, Alexander Younsi, Filippo Paggetti, Nikolai Miotk, Angelika Seitz, Martin Bendszus, Jeffrey H. Wisoff, Andreas Unterberg, Ahmed El Damaty

**Affiliations:** 1 Department of Neurosurgery, Heidelberg University Hospital, Im Neuenheimer Feld 400, 69120 Heidelberg, Germany; 2 Department of Neuroradiology, Heidelberg University Hospital, Heidelberg, Germany; 3 Department of Neurosurgery, Division of Pediatric Neurosurgery, The Hassenfeld Children’s Hospital at NYU Langone Health, New York City, NY USA

**Keywords:** CSF, Heidelberg ETV score, Endoscopic third ventriculostomy, Lamina terminalis, Third ventricular floor and ventriculoperitoneal shunt

## Abstract

In aqueduct stenosis, pressure difference below and above level of obstruction leads to bulging of third ventricular floor (TVF) and lamina terminalis (LT). Endoscopic third ventriculocisternostomy (ETV) is the standard treatment in these patients. We tried to assess success of ETV depending on those two radiological changes in aqueduct stenosis. We implemented “Heidelberg ETV score” retrospectively to assess the state of TVF as well as LT in same manner in midsagittal MR image. Every patient had a preoperative, direct, 3-months and one-year postoperative score from -2 to + 2. We correlated the scores to clinical course to decide whether the score is reliable in defining success of ETV. Between 2017–2021, 67 (mean age 25.6 ± 23.9y) patients treated with ETV were included. Success rate of primary and Re-ETVs was 91% over 46.8 ± 19.0 months. A marked shift of score to the left after surgery in success group was noticed through the distribution of score immediate postoperative, 3-months later; 70.2% showed (+ 2) before surgery, 38.9% scored (0) after surgery and 50.9% showed further score drop to (-1) 3 months later, *p* < 0.001. In cases of failure, there was initial decrease after surgery followed by increase with ETV-failure (mean time to failure: 7.2 ± 5.7 months) in 100%. Significant difference was noticed in Heidelberg score at postoperative 1-year- and failure-MRI follow-up between two groups, *p* < 0.001. Heidelberg score describes anatomical changes in third ventricle after ETV and can serve in assessment of MR images to define success of the procedure in patients with aqueduct stenosis.

## Introduction

Obstructive hydrocephalus is caused by obstruction of the cerebrospinal fluid (CSF) pathways within the ventricles [6]. A stenosis of the narrow sylvian aqueduct, which connects the third and fourth ventricles, is the most common cause of intraventricular blockage of the CSF pathways and is responsible for up to 66% of cases of hydrocephalus in pediatric and up to 49% in adult patients [6, 13, 15, 21]. The aqueduct stenosis (AS) could be a consequence of tectal tumor, brain cysts, genetic factors such as X-linked syndrome, infections, hemorrhage, or in association with CNS malformations such as spina bifida as well as many other pathologies [1, 9, 15, 20]. However, around 75% of AS cases are idiopathic [15].

Endoscopic third ventriculocisternostomy (ETV) and extracranial shunt systems such as ventriculoperitoneal shunt (VPS) are used for treating occlusive hydrocephalus [6]. ETV has become the standard treatment for obstructive hydrocephalus and is preferred over VPS [4, 8, 12, 17], due to its lower complication and revision rates, and higher long-term success rate [14, 29]. However, failure rate of ETV is not negligible and varies between 26 and 40% according to various studies [8, 14, 17]. To predict which patients would benefit from ETV, Kulkarni et al. developed the ETV success score (ETVSS) which takes into account the age of patients, etiology of hydrocephalus, and whether a previous shunt system exists, without considering preoperative imaging [16]. Additionally, the bulging of the TVF observed on preoperative MR images has been shown to be relevant in patient selection for ETV as a treatment method for occlusive hydrocephalus [2, 10, 27].

Studies have shown that the Evans ratio, third ventricle index, cella media index, and ventricular score all decrease after successful ETV [22]. However, the extent of ventricular size reduction is inversely proportional to the duration and severity of symptoms before surgery [23]. Nevertheless, lack of significant ventricular size reduction after ETV does not necessarily mean that the procedure was unsuccessful. In some cases, ventricular size may not decrease much after ETV, especially with chronic hydrocephalus, but the patient may still experience improvement in their symptoms [7]. This is because ETV can relieve pressure on the brain, even if it does not significantly reduce the size of the ventricles.

In cases of aqueduct stenosis, due to a difference in pressure between the CSF spaces above the level of obstruction (supratentorial; lateral and third ventricles) and below level of obstruction (infratentorial; fourth ventricle and basal cisterns), the third ventricular floor (TVF) bulges into the interpeduncular cistern and the lamina terminalis (LT) bulges into the suprachiasmatic cistern, which are easily visible and reflect the pressure gradient above and below the level of obstruction [25]. The susceptibility of the fine walls of these two boundaries to pressure changes leads to their shape deformity, but as they are not surrounded by neural structures, their bulging can be easily visualized [5, 6].

To date, there has been no valid method for objectively assessing these radiological signs. This single-center retrospective study is the first to evaluate preoperative, postoperative, 3-month, and one-year midsagittal Constructive Interference in Steady State (CISS) MR images using a novel score that takes into account the two radiological signs of TVF and LT bulging. We believe this score may replace the known radiological hydrocephalus indices especially following ETV as they are still no accurate in describing success of ETV.

## Methods

All patients who underwent ETV as a treatment for occlusive hydrocephalus due to sylvian aqueduct stenosis between 2017 and 2022 were included in our single-center retrospective study. Patients who had previously received VPS were excluded. This study was performed in line with the principles of the Declaration of Helsinki. Approval was granted by the Ethics Committee of University (Nr. S-084/2022), and informed consent was obtained from all patients or their parents/authorized caregivers. For all patients, MRI with CISS sequence was performed before surgery, as well as one day, three months, and one year after surgery to assess the sufficiency of the ETV. Failure of ETV was defined as the need for a Re-ETV or VP shunt insertion due to recurrence of symptoms and/or re-enlargement of the ventricles in MR imaging during follow-up. The ETV was considered a success if the patient did not require VP shunt placement during follow-up.

To determine the success of the ETV procedure, we analyzed the structure of the third ventricle floor (TVF) and lamina terminalis (LT) in the mid-sagittal MRI CISS image before surgery, as well as one day, three months, and one year after surgery. A score was calculated based on the appearance of these structures, with bulging TVF and LT assigned + 1 point each, straight assigned 0 points, and retracted assigned -1 point (see Table 1). Therefore, each patient received a score ranging from -2 to + 2, with + 2 indicating the highest-pressure gradient among the level of obstruction. The score was then correlated with the clinical course of the patients. ETV failure was defined clinically as the development of new symptoms requiring Re-ETV or insertion of a VP shunt at a later time. Additionally, the same score was used to assess radiological success, with a score of 0 indicating equalized pressure between the ventricles and basal cisterns. Patients who showed failure during follow-up were counselled regarding both options; Re-ETV versus VP shunt insertion taking into consideration the possible re-failure of ETV as well as possible shunt complications and were managed according to patient’s preference.

**Table 1:** Heidelberg score calculation

Sign	bulging	straight	retracted
Third ventricular floor (TVF)	+ 1	0	-1
Lamina terminalis (LT)	+ 1	0	-1

To monitor the changes in the anatomy of the third ventricle after surgery, we observed the direct postoperative MRI and subsequent MRIs at 3 months and one year after surgery. While the immediate postoperative MRI changes could be attributed to the release of pressure during the neuroendoscopy maneuver via CSF release, maintaining the score or even further drop after 3 months could only occur if the ETV was functioning properly.

To assess the effectiveness of the score in detecting hydrocephalic changes, we included magnetic resonance images from 67 healthy individuals without hydrocephalus who were matched to our study population.

### Statistical analysis

Continuous variables were reported as mean and standard deviation. Independent t-tests were used for intergroup comparisons of continuous variables. Heidelberg score changes in the MRI before and after surgery were compared using Fisher’s exact test. Two-sided level of significance was set at 0.05. Receiver Operating Characteristic (ROC) analysis was done between study and control groups to define Area Under the Curve (AUC), specificity and sensitivity of Heidelberg ETV score. Statistical analyses were performed using SPSS (v28.0, IBM-Corp, Armonk, NY, USA).

## Results

### Patients’ characteristics

**Fig. 1:**
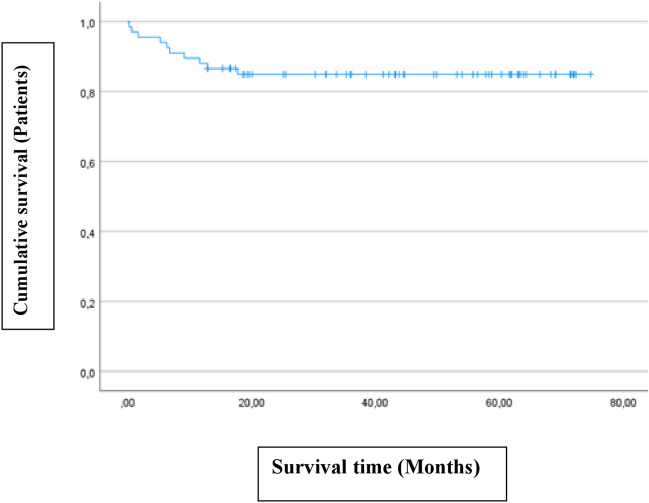
This Kaplan–Meier-Curve shows that ETV-failure occurred during medium-term follow up (Average: 7.2 ± 5.7, range: 0.27–17.8 months) after surgery. While most of patients remain asymptomatic over long term (Follow-up in mean: 46.8 ± 19.0, range: 12.9–75.9 months)

**Fig. 2:**
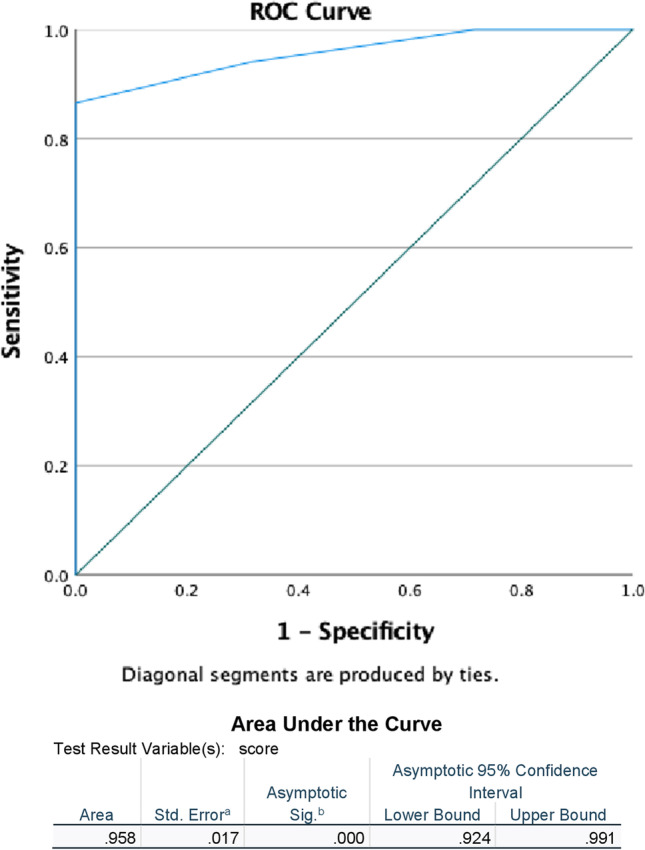
ROC Analysis of Heidelberg score data comparing study and control cohorts for detection of hydrocephalus showing AUC of 0.958 representing excellent performance

In the period between 2017 and 2022, we initially enrolled 67 patients who underwent ETV for the treatment of occlusive hydrocephalus. The study population included 28 (41.8%) with idiopathic aqueduct stenosis (iAS), 26 (38.8%) patients with tumor-related hydrocephalus, 6 (9.0%) with hydrocephalus associated with Chiari malformation, 5 (7.5%) with Blake’s pouch cyst and 2 (3.0%) with post-hemorrhagic hydrocephalus (PHH). Patients with prior VP shunt were excluded from the study assuming they have already normalized pressure gradient. Of these patients, 10 (14.9%) represented the failure group (FG), as they experienced ETV failure (5 with tumor related hydrocephalus, 2 iAS and 2 associated with Chiari malformation and one with Blake’s pouch cyst). Among them, 4 patients underwent Re-ETV (40%) and remained shunt-free, while 6 patients required VP shunt insertion (60%) during follow-up. The average time to failure was 7.2 ± 5.7 months, ranging from 0.27 to 17.8 months. The mean age of the study population was 25.6 ± 23.9 years (range 0.32–77.6 years), with 41.8% being male. All patients underwent surgery by the same surgeon, with a mean surgery duration of 55.9 ± 20.8 min (range 25.0–155.0 min, e.g. in case of performing ETV and a tumor biopsy). The mean follow-up period was 46.8 ± 19.0 months (range 12.9–75.9 months). Table 2 provides detailed information about the patients' characteristics. Figure 1 presents the Kaplan–Meier curve, showing the ETV cumulative survival during follow-up. In terms of accuracy, sensitivity and specificity, our findings indicate after ROC analysis that the Heidelberg score exhibited an accuracy of 96%, a sensitivity of 93.5%, and a specificity of 100% with AUC of 0.958 representing excellent performance. See Fig. 2.

**Table 2:** Patients’ characteristics

Variable	Patients, *n* = 67	SG, *n* = 57	FG, *n* = 10	*P*-value
Sex				
Male	28 (41.8)	24 (42.0)	4 (40.0)	1.0
Female	39 (58.2)	33 (57.9)	6 (60.0)	
Age* in years	25.6 ± 23.9	27.2 ± 23.8	16.9 ± 23.8	0.214
Follow-up* in months	46.8 ± 19.0	46.5 ± 19.5	48.5 ± 16.7	0.760
Pediatric cases	35 (50.3)	28 (49.1)	7 (70.0)	0.310
Operative time* in minute	55.9 ± 20.8	53.3 ± 14.2	70.7 ± 40.4	**0.013**
Indication				
Idiopathic Aqueduct stenosis	28 (41.8)	26 (45.6)	2 (20)	0.290
Tumor-related hydrocephalus	26 (38.8)	21 (36.8)	5 (50)	
Chiari malformation	6 (9.0)	4 (7.0)	2 (20)	
Blake’s pouch cyst	5 (7.5)	4 (7.0)	1 (10)	
Posthemorrhagic hydrocephalus	2 (3.0)	2 (3.5)	0 (0)	
3-Months-clinical Benefit	58 (86.6)	53/54 (98.2)	5/9 (56.6)	** < 0.001**
Complication rate	3 (4.5)	1 (1.75)	2 (20.0)	0.056
Total Success rate	61/67 (91.0)			
Primary ETV success rate	57/67 (85.1)			
Re-ETV success rate	4/4 (100)			
VP-Shunt implantation	6/67 (9)		6 (9)	
Time to failure* in months			7.2 ± 5.7	
Preoperative Score	*n* = 67 (100)	*N* = 57 (100)	*N* = 10 (100)	
∑2	48 (71.6)	40 (70.2)	8 (80.0)	0.433
∑1	10 (14.9)	10 (17.5)	0 (0.0)	
∑0	5 (7.5)	4 (7.2)	1 (10.0)	
∑-1	4 (6.0)	3 (5.3)	1 (10.0)	
∑-2	0 (0.0)	0 (0.0)	0 (0.0)	
Postoperative Score	*n* = 64 (95.5)	*n* = 54 (94.7)	*n* = 10 (100)	
∑2	2 (3.1)	1 (1.9)	1 (10.0)	0.555
∑1	14 (21.9)	12 (22.2)	2 (20.0)	
∑0	24 (37.5)	21 (38.9)	3 (30.0)	
∑-1	20 (31.3)	17 (31.5)	3 (30.0)	
∑-2	4 (6.3)	3 (5.6)	1 (10.0)	
3- Months Score	*n* = 63 (94.0)	*n* = 53 (93.0)	*n* = 10 (100)	
∑2	3 (4.8)	0 (0.0)	3 (30.0)	**0.002**
∑1	5 (7.9)	3 (5.3)	2 (20.0)	
∑0	21 (33.3)	18 (34.0)	3 (30.0)	
∑-1	29 (46.0)	27 (50.9)	2 (20.0)	
∑-2	5 (7.9)	5 (9.4)	0 (00.0)	
One-year vs failure Score	*n* = 56 (83.6)	*n* = 46 (80.7)	*n* = 10 (100)	
∑2	4 (7.1)	2 (4.4)	7 (70.0)	** < 0.001**
∑1	4 (7.1)	4 (8.7)	2 (20.0)	
∑0	12 (21.4)	10 (21.7)	0 (0.0)	
∑-1	23 (41.1)	19 (41.3)	1 (10.0)	
∑-2	13 (19.4)	11 (23.9)	0 (00.0)	

(%) Data in parenthesis are percentages, *Data are given as mean ± standard deviation

*SG* Success group, *FG* Failure group, ∑ the sum of Third ventricle floor (TVF) and Lamina terminals (LT)

Texts in bold font style signifies the *p* value

During the follow-up period, we observed three cases of postoperative complications (4.5%), specifically a wound healing disorder, acute subdural bleeding, and CSF leak, which required revision. At the 3-month postoperative mark, 92.1% of all patients reported being free of symptoms or showed significant clinical improvement. Further details can be found in Table 2.

Our control group of 67 patients without hydrocephalus showed in 19 patients Heidelberg score of -2, 27 patients showed score of -1 and the rest (n = 21) showed score of 0 and no patients showed any positive values which prove the fact that the score is very sensitive in detecting hydrocephalus.

### Success and failure intergroups comparison concerning pre and postoperative radiological changes

Regarding successful cases (85.1%, n = 57, including 26 iAS, 21 tumor-related hydrocephalus, 4 Chiari malformations, 4 Blake’s pouches cysts and 2 PHH), the Heidelberg score was initially + 2 and + 1 in 70.2% and 17.5% of the patients, respectively. After the surgery, the Heidelberg score decreased to 0 in 38.9% of the cases and to -1 in 31.5%. During the midterm MRI-follow-up (3 months after ETV), 50.9% of the patients experienced a drop in the Heidelberg score to -1 and 34.0% to 0. The shift in Heidelberg score from preoperative to postoperative, midterm MRI and one-year control was statistically significant with a p-value of 0.001. After one year, 73.0% of the patients showed a Heidelberg score of -1 or 0.

**Fig. 3:**
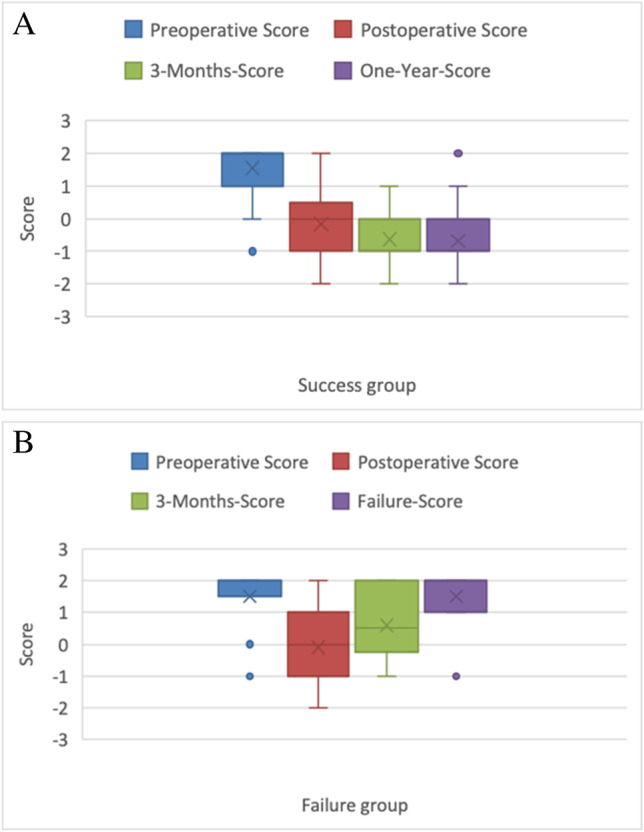
Graphical presentation of score development preoperatively, postoperatively, after three months and one year or with failure during follow-up. A highly significant difference was noticed in Heidelberg score at the postoperative three- months, one-year- (**A**) versus failure-MRI during follow-up (**B**), *p* = 0.002 and *p* < 0.001, respectively

Regarding cases of failure (n = 10, 14.9%), the Heidelberg score was initially + 2 in 8 patients (80%). After the surgery, the Heidelberg score decreased in 70% of the cases to 0, -1, or -2. However, during the follow-up MRI, which was done when failure was suspected due to recurrence of symptoms, the Heidelberg score re-increased to + 2 in 70% and to + 1 in 20%. This trend was observed during the 3-months-MRI where 50% of the failure group showed a Heidelberg score of + 2 or + 1 but were mostly not symptomatic yet. However, in the 4 patients who received Re-ETV, the score dropped from + 2 in 3 out of 4 patients back to -1 and -2 in 3 and 12 months after surgery respectively denoting radiological success. More details can be found in Fig. 3.

**Fig. 4:**
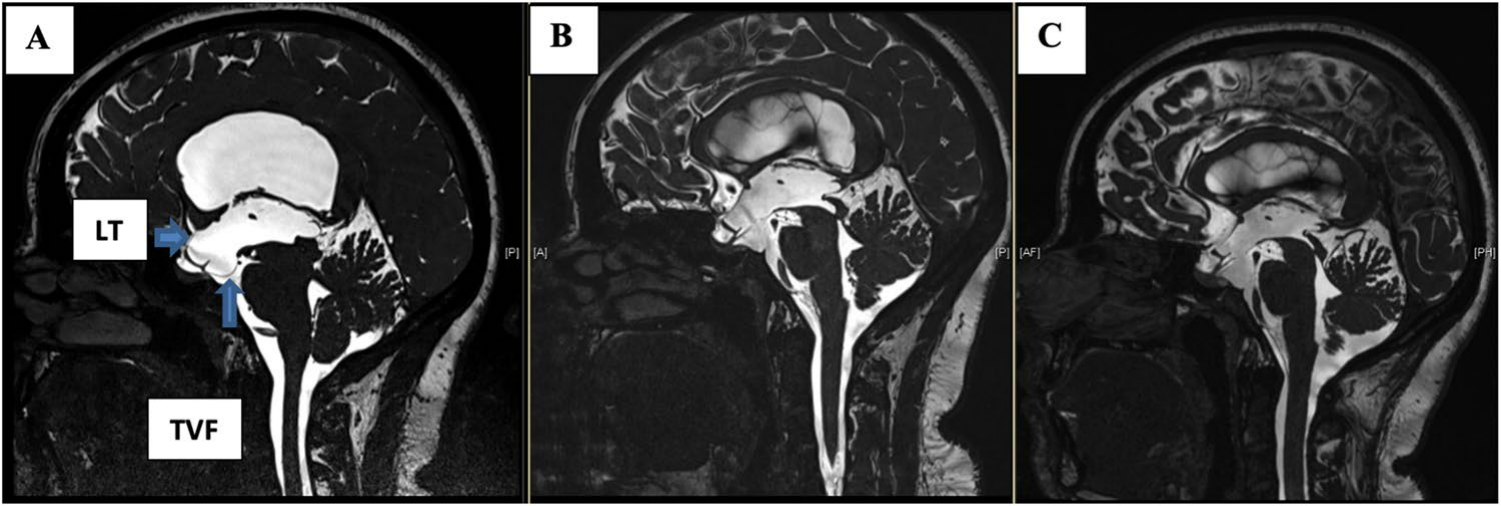
Pre-, postoperative and 3-months mid-sagittal CISS* magnetic resonance images of a 27 year old patient, who showed a preoperative Heidelberg score of + 2 (TVF** + 1 and LT*** + 1) (**A**), postoperatively, the TVF and LT went to a grade of 0 each, thus the postoperative Heidelberg score was 0, as seen in the middle image (**B**). After three months, the LT showed a reduction such that the Heidelberg score decreased to -1 as shown in the right image (**C**)

**Fig. 5:**
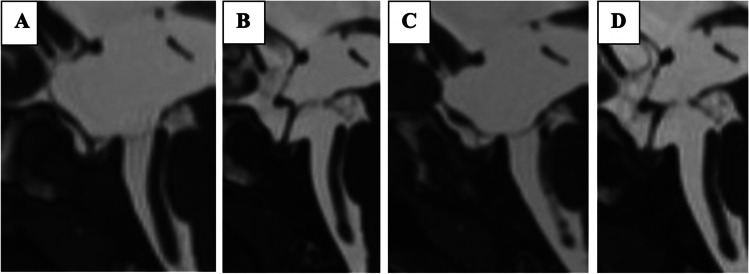
Pre-, 3-months and failure mid-sagittal CISS* magnetic resonance images of a 2 years old patient, who showed a preoperative Heidelberg score of + 2 (TVF** + 1 and LT*** + 1) (**A**), postoperatively 3 months later, the Heidelberg score went to -1 (TVF -1 and LT 0) (**B**), after 9 months, the patient showed hydrocephalic symptoms again and the Heidelberg score increased to + 2 (TVF + 1 and LT + 1) (**C**). 1 year after Re-ETV score dropped back to -1 (TVF -1 and LT 0) (**D**). *Constructive interference in steady state ** Third ventricle floor ***Lamina terminals

There was no significant difference observed in the preoperative and immediate postoperative Heidelberg score changes between the success and failure groups (p-value of 0.433 and 0.555). However, a highly significant difference (*p* = 0.002) was observed in the Heidelberg score at 3 months in the success group (SG) compared to the failure group (FG). Additionally, at one-year, a significant difference was observed in the SG and the MRI at the time of failure in the FG (*p* < 0.001). For a summary of the results, please refer to Table 2, while examples of success and failure score values are shown in Figs. 4 and 5, respectively.

### Adult group (AG) versus pediatric group (PG)

The study comprised a total of 67 patients who underwent ETV, 32 were adults (47.8%, with a mean age of 47.1 ± 16.5 years) and 35 were children (52.2%, with a mean age of 6.0 ± 5.4 years). There was no significant difference in terms of gender, operative time, follow-up duration, and complication rate between the two age groups. A rapid increase in head circumference was the most common preoperative symptom in the pediatric group (34.3%), while adults presented with headaches as the most common preoperative symptom (75.0%), and the difference was statistically significant (*p* = 0.001). For more details refer to Table 3.

**Table 3:** Comparison between pediatric (PG) and adult group (AG)

Variable	AG, *n* = 32 (47.8)	PG, *n* = 35 (52.2)	*P*-value
Gender			
Male	13 (40.6)	15 (42.9)	1.0
Female	19 (59.4)	20 (57.1)	
Age* in years	47.1 ± 16.5	5.95 ± 5.42	** < 0.001**
Follow-up* in months	47.0 ± 18.2	46.6 ± 20.0	0.932
Operative time* in minute	52.0 ± 15.8	59.4 ± 24.2	0.146
Prior ETV	4 (12.5)	0 (0)	N/A
Indication			
Idiopathic Aqueduct stenosis	17 (53.1)	11 (31.4)	
Tumor-related hydrocephalus	12 (37.5)	14 (40.0)	
Chiari malformation	1 (3.1)	5 (14.3)	0.203
Blake’s pouch cyst	2 (6.3)	3 (8.6)	
Posthemorrhagic hydrocephalus	0 (0.0)	2 (5.7)	
3-Months-clinical Benefit	31 (96.9)	27 (77.1)	0.196
Complication rate	1 (3.1)	2 (5.7)	1.0
Success rate	29 (90.6)	28 (80.0)	0.310
Preoperative Score	*N* = 32 (100)	*N* = 35 (100)	
∑2	24 (75.0)	24 (68.6)	0.239
∑1	6 (18.8)	4 (11.4)	
∑0	2 (6.3)	3 (8.6)	
∑-1	0 (0.0)	4 (11.4)	
∑-2	0 (0.0)	0 (0.0)	
Postoperative Score	*n* = 29 (90.7)	*n* = 35 (100)	
∑2	1 (3.5)	1 (2.9)	0.820
∑1	5 (17.2)	9 (25.7)	
∑0	13 (44.8)	11 (31.4)	
∑-1	8 (27.6)	12 (34.3)	
∑-2	2 (6.9)	2 (5.9)	
3- Months Score	*n* = 29 (90.7)	*n* = 34 (97.1)	
∑2	0 (0.0)	3 (8.8)	0.308
∑1	2 (6.9)	3 (8.8)	
∑0	13 (44.8)	8 (23.5)	
∑-1	12 (41.3)	17 (50.0)	
∑-2	2 (6.9)	3 (8.8)	

Texts in bold font style signifies the *p* value

The preoperative, postoperative, and 3-month Heidelberg scores did not show significant differences between the AG and PG, as shown in Table 3. Notably, 41.2% of pediatric patients achieved a TVF retraction (-1) and 47.1% achieved TVF flattening (0) 3 months after surgery. On the other hand, 82.8% of adult patients achieved LT flattening (0), *p* = 0.009. In contrast, 41.4% of adults achieved LT retraction (-1), and 55.2% achieved LT flattening (0) in the 3-month MRI-follow-up, whereas 47.1% of pediatric patients achieved LT flattening (0) and only 32.4% achieved LT retraction (-1), with 20.6% even retaining LT bulging, *p* = 0.124. Please refer to Table 3 for further details.

## Discussion

The evaluation of MRI images after surgery and during follow-up is essential for advising patients post-surgery. The Heidelberg score appears to be a valuable tool in assessing the anatomical changes in the third ventricle and determining the success of ETV. Our study demonstrated a significant shift in the Heidelberg score from preoperative to postoperative and at the 3-month MRI follow-up (*p* = 0.001). Out of the 67 patients, 10 (14.9%) exhibited recurrence of symptoms of hydrocephalus after an average of 7.2 ± 5.7 months from the initial ETV within the follow-up period (43.5 ± 16.7 months) and required re-operation with either Re-ETV or VP shunt. Thus, the success rate of primary ETV in our study was 85.1%. In the failure group, there was a significant increase in the Heidelberg score on the failure-MRI when the patients demonstrated recurrence of symptoms similar to those before the ETV surgery, indicating failure, (*p* < 0.001).

We demonstrated a comparable or even lower complication rate of 4.5% in ETV, which is in line with the reported literature rates ranging between 5 and 15% [3]. The success of ETV is measured by the absence of symptoms and the avoidance of a shunt after surgery. Secondary ETV appears to be more effective in maintaining patients shunt-free in the long term [28]. The efficacy of primary ETV has been reported to range between 36.5% and 70.6%, and of secondary ETV between 74.1% and 76%, depending on the different centers, age groups, and etiologies [28]. Recent studies have reported varying outcomes of Re-ETV in cases of failure, but overall they tend to favor its performance [19, 24]. Preferably, an endoscopic exploration that involves reopening the ventriculostomy orifice should be considered as a treatment option. Our study only included cases of primary and Re-ETV, while cases of secondary ETV with prior VP shunt presented with dysfunction were excluded. Among the patients included in our study, primary (57/67) and Re-ETV (4/4) showed an efficacy of 91% (61/67).

Several studies have focused on different aspects of ETV to determine its efficacy and success rate. While some studies have concentrated on preoperative MR imaging [2, 10, 11, 26, 27], others have relied on the patient's clinical information [16, 18]. Kulkarni et al. developed the ETV Success Score (ETVSS) to predict the 6-months success rate of ETV in pediatric patients with hydrocephalus, based on age, etiology and presence of a previous shunt [16, 18]. Another study conducted by Wang et al. focused solely on the sign of third ventricle floor bowing in preoperative MRI as a predictor of ETV success. The study included 42 hydrocephalic infants (< 1-year-old) within five years. All infants showed this sign on preoperative imaging, and the ETV success rate was 71.4% [26]. Dlouhy et al. [10] classified depression of the third ventricular floor and anterior curvature of the lamina terminalis on MR imaging by 59 ETVs. They found that lack of either lamina terminalis, supraoptic recess, or third ventricular floor preoperative bowing was significantly associated with ETV failure, with just 33% of patients without bowing of the anterior third ventricular treated successfully with ETV [10]. The Heidelberg score used in our study relies on the dynamic change in preoperative, postoperative, 3-months and 12-months imaging, and it can be used to expect ETV success through a high (+ 2) preoperative score, as well as to prove radiological success observed with a decline in Heidelberg score on MR images following surgery. Considering that an improvement in the known hydrocephalus parameters; Evans ratio, third ventricle index, cella media index, and ventricular score is not always noticed following ETV, especially in chronic hydrocephalus, although the procedure succeeded in relieving the elevated pressure and alleviating the patients’ symptoms, we believe that our suggested Heidelberg ETV score would be of a great value in assessment of radiological success during follow-up.

In our study, we observed no significant differences between the pediatric and adult patient groups regarding patient numbers, complication rates, and the preoperative, postoperative, three-month, and one-year Heidelberg score values, suggesting that the Heidelberg score can be reliably used across all age groups with occlusive hydrocephalus. However, we did observe that the most significant retraction after three months of ETV occurred in the pediatric group in the third ventricle floor and in the adult group in the lamina terminalis. This difference may be attributed to anatomical variations, thickness, and rigidity of these structures that can vary with age and duration of exposure to elevated pressure. Further research is needed to investigate and elucidate this phenomenon through prospective studies.

The study has some limitations, such as its retrospective nature and the modest number of cases analyzed, which may limit its generalizability. Also, we could not use the Heidelberg score to “predict” before surgery who would fail but we were able to detect a pattern of score drop in cases of success where a further drop 3 months after ETV was considered a good indicator of long term success compared to cases of failure where the score started to increase again at 3 months or at least showed no further drop. Unfortunately, due to the modest number of cases analyzed we could not prove that pattern statistically. Therefore, further prospective studies, including more patients with a broader range of etiologies, are necessary to confirm the validity of the proposed Heidelberg score. Nevertheless, once validated, the Heidelberg score has the potential to be a useful tool in predicting the long-term success rate of ETV in hydrocephalic patients based on changes in the score after surgery.

## Conclusion

The Heidelberg score is based on the changes in the anatomy of the third ventricle observed on MR imaging before and after ETV treatment, including preoperative, postoperative, three-month, and one-year scans. A significant decrease in the score was observed in the first three months, which can be interpreted as an indication of a reduction in the volume of the third ventricle and a functioning ETV. Our findings indicate that the Heidelberg score exhibited an accuracy of 96%, a sensitivity of 93.5%, and a specificity of 100% with AUC of 0.958 representing excellent performance. Hence, this score is a reliable tool for assessing ETV success from a radiological standpoint. However, more studies with longer follow-up periods comparing successful and failed ETV cases are necessary to confirm its efficacy.

## Data Availability

Not applicable.
